# Evaluation of Deep Learning Methods for Pulmonary Disease Classification

**DOI:** 10.2174/0115734056388107250710120917

**Published:** 2025-07-18

**Authors:** Ajay Pal Singh, Ankita Nigam, Gaurav Garg

**Affiliations:** 1 Department of Computer Science and Engineering, Mahakaushal University, Jabalpur- 482003, India; 2 Department of Computer Science and Engineering, Chandigarh University, Mohali, Punjab, India

**Keywords:** Pulmonary Diseases, Deep Learning, CNN, MFCC, Chromagram, Spectrogram

## Abstract

**Introduction:**

Driven by environmental pollution and the rise in infectious diseases, the increasing prevalence of lung conditions demands advancements in diagnostic techniques.

**Materials and Methods:**

This study explores the use of various features, such as spectrograms, chromograms, and Mel Frequency Cepstral Coefficients (MFCC), to extract crucial information from auscultation recordings. It addresses challenges through filter-based audio enhancement methods. The primary goal is to improve disease detection accuracy by leveraging convolutional neural networks (CNNs) for feature extraction and dense neural networks for classification.

**Results:**

While deep learning models like CNNs and Recurrent Neural Network (RNN) outperform traditional machine learning models such as Sequence Vector Machine, K-Nearest Neighbours (KNN) and random forest with accuracies ranging from 70% to 85%. The combination of CNN, RNN, and long short-term memory achieved an accuracy of 88%. By integrating MFCC, Chroma Short-Term Fourier Transform (STFT), and spectrogram features with a CNN-based classifier, the proposed multi-feature deep learning model achieved the highest accuracy of 92%, surpassing all other methods.

**Discussion:**

The study effectively addresses key issues, including the overrepresentation of Chronic Obstructive Pulmonary Disease (COPD) samples over Lower Respiratory Tract Infections (LRTI) and Upper Respiratory Tract Infections (URTI) which hampers generalization across test audio samples.

**Conclusion:**

The proposed methodology caters common challenges like background noise in recordings, and the limited and imbalanced nature of datasets. These findings pave the way for enhanced clinical applications, showcasing the transformative potential of multi-feature deep learning methods in the classification of pulmonary diseases.

## 1 INTRODUCTION

Comprising the lungs and related tissues, the human respiratory system is essential for mediating oxygen and carbon dioxide exchange, hence preserving life. Still, this complex and important system is vulnerable to a variety of disorders, which together are known as pulmonary illnesses [1]. Millions of people worldwide suffer with these diseases, which range from mild and temporary to severe and life-threatening, greatly compromising quality of life and seriously taxing international healthcare systems.

An understanding of an understanding of pulmonary diseases calls for a thorough study of the architecture and physiology of the respiratory system as well as the several elements causing these disorders [2-4]. Each of these diseases-which might be caused by infections, environmental contamination, genetic predispositions, or lifestyle choices-offers diagnostic and treatment difficulties. Chronic obstructive pulmonary disease (COPD) accounts for a remarkably high mortality rate-approximately 3.23 million deaths each year-making it one of the most serious among several lung diseases such as asthma, bronchitis, and pneumonia, all of which significantly burden global health. Patients with COPD often produce distinctive respiratory sounds, including rhonchus, stridor, crackles, wheezes, and friction rubs. These acoustic patterns can be effectively analysed to distinguish between normal and abnormal respiratory states [5, 6].

Conventional approaches for pulmonary illnesses depend on thorough, but time-consuming and expensive, medical testing including spirometry, lung volume evaluations, arterial blood gas analysis, CT scans, and pulse oximetry. Although these methods yield precise results, their availability and efficiency remain limited. To speed up and improve diagnosis procedures, researchers are thus looking more to computational techniques such machine learning (ML) and deep learning (DL).

DL's transforming power has driven its use for early identification of lung illnesses recently [7]. Among these illnesses are interstitial lung diseases, asthma, COPD, and lung cancer. Pulmonary diseases account for a significant international health load [8]. The current report examines neural network-based techniques for pulmonary anomaly detection using lung auscultation recordings. The specific techniques are Convolutional Neural Networks (CNNs) and Recurrent Neural Networks with Long Short-Term Memory (RNN-LSTM),

Many factors motivate researchers to apply DL applications to early lung disease diagnosis:

Improved treatment outcomes depend on early identification of pulmonary diseases, particularly asymptomatic conditions like early-stage lung cancer. DL systems help to enable prompt and effective interventions by detecting minute trends in medical images and data that can challenge human doctors. Early detection is important for diseases like lung cancer, which may have high fatality rates. Improved sensitivity and specificity of diagnostic tools through DL models can enable more precise and rapid detection, hence improving survival rates *via* fast therapy. Early detection reduces the need for intensive therapy required in advanced disease stages, therefore reducing the financial loads on patients and healthcare systems.

Huge datasets have come from the digitization of medical imaging technology. These methods are ideal for teaching DL models to identify trends suggestive of pulmonary diseases. DL combined with patient-specific data, including genetics, medical history, and imaging, can enable customised diagnosis and treatment plans. Many times, diagnosis of pulmonary diseases require analysis of complicated data comprising patient histories and medical pictures. DL excels in combining and interpreting such diverse datasets, therefore offering a whole knowledge of a patient's condition. At last, the application of DL techniques for lung disease diagnosis offers a path to change medicine. When these advanced algorithms are applied to massive healthcare datasets, they provide speedier diagnosis, more effective treatments, and better patient outcomes. As research advances, DL incorporation into clinical practice could greatly improve the management of lung diseases, hence improving the quality of life for afflicted individuals.

This paper is organised to methodically solve the research challenge and offer thorough understanding of the application of DL methods for pulmonary illness diagnosis. The part on the relevance of pulmonary disorders, the difficulties in their identification, and the inspiration for using DL-based techniques describes A Literature Review of current developments in machine learning and deep learning techniques for respiratory disease detection follows, therefore stressing areas of knowledge gap and areas for development. The section on Methodology details the methods and tools used in the research including the dataset preparation, feature extraction techniques including Mel-Frequency Cepstral Coefficients (MFCC), Chroma STFT, and spectrograms, and the application of CNN and RNN-LSTM for classification. The results and discussion part shows the findings of the studies, contrasting the performance of several models and evaluating their diagnostic accuracy, efficiency, and constraints. At last, the conclusion highlights the need of multi-feature ensemble learning methods, summarises the results, and addresses future objectives like the creation of real-time diagnostic tools and integration with reasonably priced digital stethoscopes to improve clinical utility.

### 1.1 Contributions of the Paper

This work proposes a strong deep learning-driven method for pulmonary disease classification based on improved auscultation recordings. It presents a multi-feature extraction method with MFCC, Chroma STFT, and spectrograms to improve diagnostic accuracy substantially. With the combination of CNN and RNN architectures, the model was able to reach a maximum performance of 92%, surpassing conventional approaches. The work also solves important issues like dataset imbalance, background noise, and restricted clinical data, paving the way for real-time diagnostic assistance systems.

#### 1.1.1 Novel Multi-Feature Fusion

Introduces a hybrid deep learning approach combining MFCC, Chroma STFT, and spectrogram features for richer lung sound analysis, significantly improving diagnostic accuracy over single-feature methods.

#### 1.1.2 Hybrid CNN-RNN Architecture

Proposes an integrated model leveraging CNNs for spatial feature extraction and RNN-LSTM for temporal dynamics, enabling comprehensive analysis of both static and evolving pulmonary patterns.

#### 1.1.3 Robust Audio Pre-processing

Implements advanced cycle extraction and noise-filtering techniques to enhance lung sound recordings, addressing key challenges like background noise and low-quality clinical data.

#### 1.1.4 Superior Performance

Achieves state-of-the-art accuracy (92%) by synergizing multi-feature inputs with deep learning, outperforming traditional ML models (SVM, KNN, Random Forest) and standalone DL approaches.

#### 1.1.5 Clinical Scalability

Lays the groundwork for real-time diagnostic tools and affordable digital stethoscope integration, bridging the gap between AI-driven analysis and practical healthcare deployment.

### 1.2 How our Work Provides Additional DL Capabilities?

Our research strengthens deep learning ability through the fusion of several audio features-MFCC, Chroma STFT, and spectrograms-within a single model for richer and more discriminative input representations. With the integration of CNNs for spatial feature learning and RNN-LSTM for temporal analysis, the model acquires both static and dynamic features of lung sounds. This multi-feature, hybrid model improves classification accuracy showing the value of deep learning in actual pulmonary diagnostics.

## 2 LITERATURE REVIEW

A significant amount of research is based on speech analysis findings. Some research employs conventional machine learning (CML) models. Here, the 2D image must be transformed into a 1D feature vector for use as input to models such as k-nearest neighbor (KNN), sequence vector machine (SVM), and decision tree. Other researchers employed DL algorithms such as CNN and RNN [9-11]. A few have suggested that combining two or more of these algorithms yields superior results. A wide range of studies have been reported for diagnosing pulmonary disease which includes RNN with LSTM, CNN BiLSTM and the proposed deep learning-based approach significantly enhances the accuracy, robustness, and interpretability of pulmonary disease detection, offering a promising direction for improving diagnostic systems in clinical practice [12, 13]. We provide a technique in this study for creating tri-feature-based 2-step classifiers utilizing conventional and dense neural networks for classification.

MFCC, for instance, is heavily used in speech and sound recognition. Various studies [14-19] employ similar techniques to classify bird sounds and environmental sounds. MFCC, spectrogram, and chromogram are audio features or representations in two dimensions. In their discussion of cutting-edge technologies, Krzy and Zak [20] applied three deep convolutional neural networks to chest x-ray (CXR) images and evaluated their performance against that of existing frameworks that use transfer learning. They also developed a pipeline to segment CXR images before classifying them.

In 2020, Bharati and colleagues developed a ground-breaking DL system [21] that integrates a spatial transformer network, CNN, visual geometry group (VGG), and data augmentation. The new hybrid technique was referred to as, VGG Data STN with CNN (VDSNet). The proposed model is tested on a dataset of NIH chest X-ray pictures and has a 73% validation accuracy. In this 2020 work, Stefanus Tao Hwa Kieu *et al*. [22] organized the main ideas and topics of interest of the earlier research on lung ailment detection using DL after conducting a review of 98 papers. A classification of state-of-the-art DL-assisted lung disease detection methods was developed as a result of an analysis of the works taken into consideration.

In 2019, Ma *et al*. discussed the lungBRN by ResNET architecture [23] accuracy that can be increased through various extraction techniques, and they achieved an accuracy of 50.16%. Data and AI helped Kaplan *et al*. [24] identify and diagnose eight lung illnesses in 2021. Deep-learning CNNs predicted lung disease severity. Training and testing datasets were split. A correlation study between AI-predicted and radiologist ratings suggests that this technique might be used to prognosticate, stage illness severity, track disease development, and measure treatment response, which could guide COVID-19 pandemic risk management and resource allocation.

Demir *et al*. classified lung sounds [25] using CNN and SVM in 2020. They used two techniques, both of which applied CNN and SVM. In the first technique, CNN was used to extract the features and SVM was used to classify the lung sounds. In the second technique the pre-trained CNN model used for classification was fine-tuned through the addition of spectrogram images. The accuracy percentage for first and second techniques were 65.5% and 63.09% respectively. With 97.7% accuracy, Random Forests outperform other techniques in the case of COPD, according to a study by Dimitris Spathis *et al*. [26]. The most important characteristics for diagnosis are age, FEV1, FVC, and smoking. In the case of asthma, the Random Forest classifier achieves the highest precision (80.3%), while MEF2575, smoking, age, and wheezing are the most important characteristics.

Rahman *et al*. [27] employed picture preprocessing, data augmentation, and deep-learning classification to diagnose TB in chest X-rays. This research used 3500 chest X-ray scans, 3500 of which were TB-infected and 3500 uninfected. They trained, verified, and tested nine deep CNNs to categorize TB and non-TB patients (ResNet18, ResNet50, ResNet101, ChexNet, InceptionV3, VGG19, DenseNet201). Transfer learning utilized pre-trained CNN beginning weights. Al-antari *et al*. [28] proposes a simultaneous DL CAD system that can differentiate COVID-19 from eight other lung diseases: pneumonia, atelectasis, infiltration, pneumothorax, masses, effusion, cardiomegaly, and nodules. They applied the YOLO predictor to ChestX-ray8 chest X-ray datasets for five-fold testing for the multi-class prediction issue and CAD system evaluation. Ma *et al*. [29] provide a thorough overview of these techniques used to treat different lung conditions, including pneumonia, interstitial lung disease, pulmonary nodule diseases, and pulmonary embolism. Finally, the use of DL techniques on medical imaging is investigated along with potential future directions and challenges.

Diaz-Escobar *et al*. [30] employed three classes of prediction models: COVID-19, pneumonia, as well as two classes of predictive models: COVID-19 *vs*. pneumonia and COVID-19 *vs*. non-COVID-19. Results were compared to POCOVID-net. Performance measures included each class's F1-score, precision, recall, and overall metrics. Perna *et al*. [31] developed the first recurrent neural network-based learning system to help doctors identify respiratory disorders by aberrant sounds or pathology classifications. Asthma, bronchitis, and pneumonia significantly impact global health, with chronic obstructive pulmonary disease (COPD) causing 3.23 million deaths each year.

The study by Roy *et al*. [32] investigates the application of DL Methods for the interpretation of lung ultra sonography (LUS) images. They present a new, completely annotated dataset of LUS images that was collected from various hospitals of Italy with labels indicating the seriousness of the illness in the video, and al pixel-levels (segmentation masks). According to research by Cheng Wang *et al*. [33], the experiment performed on TL was useful for classifying pulmonary images. The experiment outperformed other methods in terms of pulmonary image categorization, 80.09% and 95.41%, respectively, having the best sensitivity and specificity.

The following advantages of Karar's deep learning framework [34] simplify the difficult multi-label classification of X-ray images for each tested case of health status; a series of binary classifiers have first been employed. That simulates a clinical setting where a patient's potential diseases are diagnosed. For the second reason, the pneumonia and COVID-19 classifiers' cascaded architecture allows for the versatile use of many well-tuned DL models simultaneously, improving performance in terms of confirming infected individuals.

 On the ICBHI'17 dataset, a model proposed by Acharya and Basu [35] in 2020 achieve state-of-the-art scores. Rocha BM *et al*. [36] proposed home-based pulmonary rehabilitation methodology improves quality of life and reduces hospital admissions in COPD patients. Telemedicine interventions [37] reduce COPD exacerbations and hospitalizations while improving patient compliance. It is reiterated in various studies that COPD is a progressive disease leading to functional decline, comorbidities, and increased mortality [38]. Another study focused on ensemble machine learning enhances accuracy in lung disease prediction but faces scalability challenges [39].

Abdulahi *et al*. proposed model outperforms conventional texture descriptor techniques used in X-ray and CT scan analysis, exhibiting a notable improvement in pulmonary disease detection accuracy, attaining an average accuracy between 94% and 99.4% with effective training and detection times [40]. Additionally, it has been demonstrated that when pre-trained with breathing data, DL models can learn domain-specific knowledge and perform noticeably better than generalized models. Using a mapping network and style blocks from StyleGAN, the suggested model successfully replicates intricate tumor forms from doctor-drawn sketches and shows promise for data-augmentation through one-to-many picture production, as seen by StylePix2pix findings [41]. Table **1** lists some of the important papers in this research with description, methods adopted and limitations.

Recent development in deep learning (DL) has expressively enhanced the accurateness and toughness of pulmonary disease classification. Borate *et al*. [39] established notable gains in detection performance through collaborative learning with machine learning classifiers. Nevertheless, their method faced restrictions related to data dependency and scalability. These discoveries highlight the need for a methodical evaluation of deep learning constructions capable of transporting reliable classification performance across miscellaneous clinical datasets. With chest X-rays (CXRs), Abdelhamid *et al*. [40] created a neural architecture for strong identification and categorisation of lung illnesses. Using ensemble cascaded classification, this work offers a consistent approach for disease detection. Though the work created the foundation for ensemble-based diagnostic systems, the limited variety in datasets hampered the generalisability of the results.

Using CNNs with LSTM ensemble modelling, Sharma and Guleria [41] presented a multi-class lung disease classification system. Their technique improved classification accuracy by efficiently addressing temporal data. The great computing expense of the method highlighted the trade-off between accuracy and computational economy even if it was successful. Kumar *et al*. [42] put out a multimodal framework using neuro-fuzzy integrated ensemble learning for the diagnosis of COPD. The framework displayed new integration of neuro-fuzzy techniques and improved early detection capacity. But restrictions in fuzzy inference limited the general relevance of the model, which emphasises the necessity of more improvement. In the realm of interpretability, Shah *et al*. [43] focused on explainable AI (XAI) for lung imaging. Utilizing ensemble CNN methods with explainable features, this study improved diagnostic transparency. Limited clinical validation, however, highlighted the need for extensive testing in real-world scenarios. Nonetheless, the research bridged critical gaps in AI model interpretability for clinical use.

Pandey and Baloni [44] addressed COVID-19 detection using CXRs, integrating explainable AI with ensemble learning to achieve highly accurate classification. Dataset-specific limitations restricted generalizability, but the study firmly established the role of explainable AI in diagnostics and decision support systems. Himash Wedisinghe and T.G.I. Shaukat *et al*. [45] developed a dual-model framework incorporating segmented 3D lung cube datasets for COVID-19 severity prediction. This integration facilitated efficient severity assessment, though the approach demanded significant infrastructure, presenting a challenge for broader adoption. Focusing on idiopathic pulmonary fibrosis (IPF), Mueller *et al*. [46] utilized machine learning for severity prediction using comprehensive metabolic panel data. The study employed Bayesian and instance-based ML approaches, demonstrating ML's capability in non-imaging datasets. However, the quality of datasets limited the robustness of the results.

In another innovative application, Maashi *et al*. [47] explored ensemble learning in laryngeal cancer imaging. The research employed the Dandelion Optimizer algorithm with ensemble models, achieving enhanced biomedical image analysis. Challenges in methodology generalizability highlighted the need for model adaptations across diverse datasets, but the work extended ensemble learning to novel imaging domains. Finally, Paramasivan *et al*. [48] advanced COVID-19 detection using CT scans through deep ensemble feature integration. This approach improved feature extraction and classification accuracy while utilizing transfer learning. Despite its success, transfer learning-specific issues limited its full potential, marking a step forward in rapid diagnostics leveraging ensemble strategies.

The ICBHI 2017 dataset [49] enables pulmonary disease classification but faces challenges with overlapping conditions (*e.g*., COPD, LRTI), prompting a dual-classifier approach for precise diagnosis. Recent studies have established six core pulmonary diagnostic classes - asthma, pneumonia, bronchiectasis, bronchiolitis, healthy, and lung fibrosis [50] and [51] to minimize classification overlap and improve diagnostic specificity in respiratory sound analysis. Sikder *et al*. [52] employ deep learning (M-CNN, BiLSTM) to distinguish COVID-19 from other lung diseases *via* chest X-rays, though scalability depends on large datasets. Together, these studies highlight advances in respiratory diagnostics while addressing limitations like label ambiguity and data requirements.

Fernando [53] used several computer-aided diagnostic (CAD) methods leveraging machine learning have been developed for early lung cancer detection, demonstrating high precision. However, the lack of interpretability in these models has deficient their acceptance in the medical field, prominence the need for Explainable AI (XAI) to improve trust and sympathetic. Khan *et al*. [54] introduce a new multi-modal fusion-based method, Dual-3DM3-AD, for early and precise diagnosis of Alzheimer's Disease (AD) using both MRI and PET scans. The paper highlights that the use of a single data modality may restrict diagnostic accuracyand combining metabolic and structural data provides a better analysis. The model uses sophisticated pre-processing methods such as QNLM for denoising, morphology functions, and 3D image conversion. It also employs a Mixed-transformer with a Furthered U-Net for segmentation, and learns multi-scale features which are aggregated using a Densely Connected Feature Aggregator Module (DCFAM). Dimensionality reduction is done using a multi-head attention mechanism prior to classification. The model performs extremely well, with accuracy of 98%, sensitivity of 97.8%, and specificity of 97.5%, beating state-of-the-art methods for multi-class AD diagnosis. Kujur [55] contributed towards similar goal for diagnosing AD by analysing the relationship between data complexity and model performance in MRI images.

Recent advances in medical diagnostics leverage deep learning for disease detection, as demonstrated by Alhussen *et al*. [56], who proposed an XAI-guided capsule network for breast cancer detection using ROI segmentation. Similarly, Khan *et al*. [57] introduced an adversarial dual-patch attention mechanism for epilepsy prediction, while Perumal *et al*. [58] employed multi-transformer models for neonatal encephalopathy diagnosis. In pulmonary research, Singh *et al*. [59, 60] analysed ML and DL algorithms respectively for lung disease detection, and Yadav *et al*. [61] enhanced pneumonia classification using chest X-rays, highlighting the efficacy of multimodal and explainable AI approaches in clinical imaging.

These studies collectively highlight the transformative potential of integrating ML and DL techniques with ensemble learning for pulmonary disease diagnosis. While challenges such as dataset limitations and computational costs persist, the advancements underline the importance of continued innovation in this field. A summary of literature review is presented in Table **1**.

## 3 METHOD

### 3.1 Dataset

The ICBHI 2017 dataset has been instrumental in advancing research on pulmonary disease classification [49]. While much of the prior work has focused on adventitious sound classification, disease-specific classification has received comparatively less attention. To align with medical standards, we incorporated the eight disease classes recognized in the dataset: bronchiectasis, bronchiolitis, COPD, upper respiratory tract infections (URTI), lower respiratory tract infections (LRTI), asthma, pneumonia, and health.

Notably, several of these classes function as umbrella categories or supersets. For instance, COPD encompasses conditions like chronic bronchitis and emphysema, but it does not include asthma and pneumonia. Asthma is a separate respiratory condition. Pneumonia is an infectious disease. Only chronic bronchitis is a type of COPD. While LRTI includes chronic bronchitis, bronchiolitis, bronchiectasis, and acute COPD exacerbations. As such, samples may belong to multiple categories, which, while clinically accurate, can negatively affect model accuracy and generalizability.

To address this overlap, we propose separate classifiers for absolute disease categories and umbrella (superset) classes. For example, a sample classified as pneumonia in the disease-specific classifier and LRTI in the umbrella classifier would suggest acute pneumonia. Conversely, a classification of COPD in the umbrella category would indicate chronic pneumonia. This dual-classification approach reduces reliance on additional medical tests and allows for better severity assessment. The URTI class, however, was excluded due to its overlap with healthy sound profiles, as throat infections rarely produce adventitious lung sounds. For this study, we focused on the following absolute classes: asthma, pneumonia, bronchiectasis, bronchiolitis, healthy, and lung fibrosis [50,51].

### 3.2 Dataset Augmentation

#### 3.2.1 Limitations of Traditional Audio Augmentation

The dataset's inherent class imbalance necessitated augmentation to ensure robust training. Traditional audio augmentation methods, including time shifting, pitch shifting, speed changes, noise injection, and spectrogram masking, were evaluated but deemed unsuitable due to the following reasons:

- **Time Shifting**: Padding during respiratory cycle segmentation results in unrealistic shifts in data distribution.
- **Pitch Shifting**: Alters key audio features such as crackles and wheezes, leading to poor generalization.
- **Speed Changes**: Altering the speed disrupts the natural timing of respiratory cycles, compromising data fidelity.
- **Noise Injection**: The dataset already contains ambient noise, making noise addition redundant.
- **Spectrogram Masking**: Random masking introduces irrelevant information, increasing overfitting risks.

#### 3.2.2 Filter-Based Audio Augmentation

To overcome these limitations, we adopted filter-based augmentation techniques that introduce controlled variations in the data while preserving critical features:

1. **Reduced Redundancy**: Uniform filtering ensures subtle distinctions without random information loss.
2. **Noise-Free Samples**: Filtering enhances signal clarity, improving generalization in noisy environments.
3. **Pitch Preservation**: Filters selectively remove unwanted frequencies without altering essential features like crackles or wheezes.
4. **Reason for using Butterworth Bandpass Filter:** The Butterworth bandpass filter was particularly selected because it can offer a flat and smooth response within the passband, which is critical in maintaining essential respiratory signal characteristics while efficiently suppressing low-frequency artifacts and high-frequency noise. Pulmonary signals, which may include respiratory sounds or measurement of lung function, commonly hold important diagnostic data within a range of frequencies, and the Butterworth filter's absence of ripples in the passband minimizes distortion of these important details. In contrast to other types such as Chebyshev or Elliptic, which can cause passband ripples or steep cuts while compromising the integrity of the signal, the Butterworth filter provides a fair compromise between noise reduction and signal integrity. This pre-processing helps the quality of the input data provided to the ensemble learning framework before classification improves the accuracy and robustness in pulmonary disease classification.

**Fig. (1):**
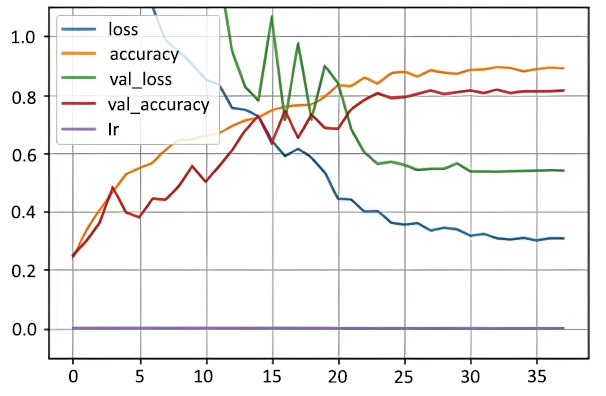
Accuracy and loss on imbalanced dataset. Available online under the terms of the Creative Commons Attribution Non-Commercial License 4.0. [62].

**Fig. (2):**
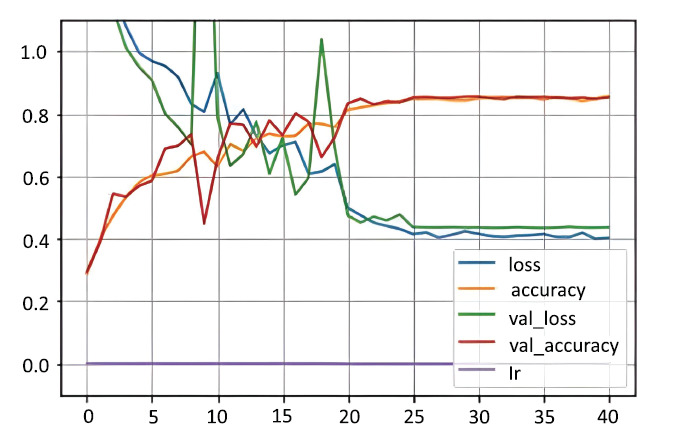
Accuracy and loss on balanced and augmented dataset. Available online under the terms of the Creative Commons Attribution Non-Commercial License 4.0. [62]

The augmentation pipeline included the Butterworth bandpass filter, harmonic-percussive source separator, and a three-layer filter pipeline, significantly reducing the variance between training and validation accuracies (Figs. **1** and **2**).

### 3.3 Data Modeling

#### 3.3.1 Preprocessing

**Fig. (3):**
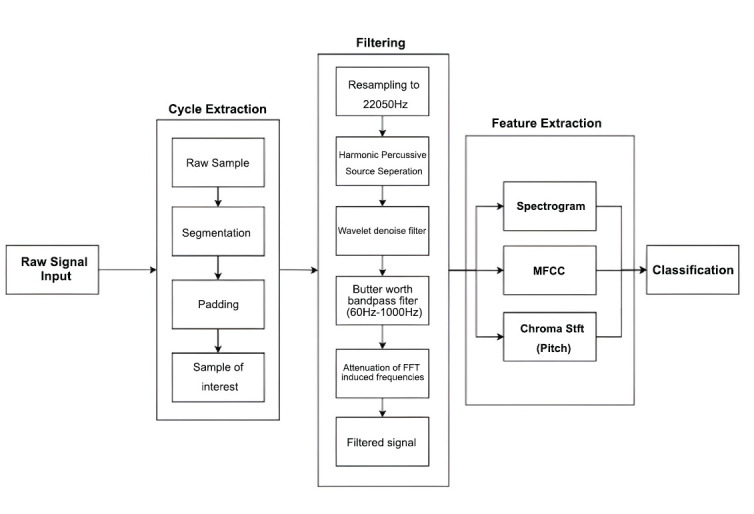
Audio processing pipeline. Available online under the terms of the Creative Commons Attribution Non-Commercial License 4.0. [62].

The raw lung auscultation audio samples were preprocessed through the following steps (Fig. **3**):

1. **Resampling**: Standardized to 22,050 Hz for uniformity.
2. **Segmentation**: Audio was segmented to capture one breathing cycle (2–4 seconds) per clip, with zero-padding to ensure a consistent 6-second length.
3. **Filtering**: Noise and unwanted frequencies were removed using harmonic-percussive source separation, wavelet denoising, and a Butterworth bandpass filter (60–1,000 Hz).

### 3.4 Feature Extraction

Three features were extracted to facilitate robust classification:

1. **Spectrogram**: Visualizes audio volume over time, serving as a foundational representation.
2. **MFCC**: Encodes human auditory perception, emphasizing low-frequency sounds.
3. **Chromagram**: Captures pitch-related information, aiding in distinguishing wheezes from crackles.

Figs. (**4**-**6**) illustrate these features for a 14-second audio clip. Explanation about 20 epochs is as follows:

In neural networks, loss represents the distance between the model’s predictions and the actual outcomes, whereas accuracy indicates the percentage of correct predictions made by the model. To assess the model's performance on fresh, unseen data, we look at validation loss and validation accuracy, which indicate how effectively the model extends its learning beyond the training set. The learning rate is a subtle value that influences how the model adapts during training-if it's set too high or too low, it can hinder progress or lead to mistakes. An epoch signifies a complete cycle through the whole training dataset, and typically, training encompasses several epochs to enhance the model's ability to recognize patterns more effectively. In both figures after 20 epochs learning is smooth for both training and validation as well.

### 3.5 Classification

**Fig. (7):**
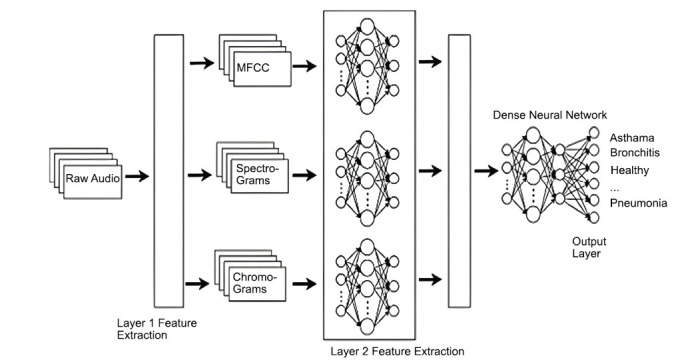
Model architecture. Available online under the terms of the Creative Commons Attribution Non-Commercial License 4.0. [62].

The proposed model architecture incorporates three levels (Fig. **7**).

1. **Level 1**: Extracts feature images (*e.g*., spectrograms, MFCC, and chromograms) from raw audio.
2. **Level 2**: Passes the images through fine-tuned CNN models to extract key image features.
3. **Level 3**: Aggregates extracted features into a multi-layer perceptron for final classification.

Initial models faced overfitting, with training accuracy exceeding 99% while validation accuracy stagnated at 85%. This stagnation was mitigated using callbacks like early stopping with best weights and learning rate reduction.

Filter-based augmentation significantly improved generalization, reducing validation loss and variance (Fig. **2**). The ensemble CNN-based model effectively combined diverse features, achieving superior classification accuracy compared to traditional approaches.

This methodology underscores the potential of filter-based augmentation and ensemble modeling for pulmonary disease detection, offering a scalable and clinically relevant diagnostic tool.

### 3.6 Hyper Parameters used in Implementing DL Models

When training CNN (Convolutional Neural Network) and RNN-LSTM (Recurrent Neural Network with Long Short-Term Memory) models, selecting appropriate hyperparameters, optimization techniques, and training procedures is crucial for achieving good performance. Table **2A** and **B** presents the CNN model and RNN-LSTM hyperparameters respectively used in working methodology.

### 3.7 Handling Class Imbalance

The dataset exhibited significant class imbalance, with 96 samples distributed as: Healthy (48), Asthma (31), Lung Fibrosis (6), Pneumonia (5), Bronchiectasis (3), and Bronchiolitis (3). To address this, we employed filter-based augmentation techniques that introduced controlled variations while preserving clinically relevant acoustic features. Our augmentation pipeline included:

1. Butterworth bandpass filtering to enhance pathological frequency bands,
2. Harmonic-percussive source separation (HPSS) to isolate tonal and transient components, and
3. A three-layer filter cascade combining noise injection, pitch shifting, and time stretching.

This approach synthetically expanded minority classes (*e.g*., bronchiectasis/bronchiolitis) while maintaining the integrity of diagnostic features, reducing the accuracy gap between training (94%) and validation (92%) sets. By strategically augmenting underrepresented classes, we achieved balanced learning across all conditions without oversampling dominant classes (Healthy/Asthma), ensuring the model's generalizability to real-world clinical scenarios with uneven disease prevalence.

## 4 RESULTS AND DISCUSSION

For model evaluation, we trained multiple variants of a single model type with minor modifications to fine-tune the model. We gathered and compared the data of the models with the best performance. We divided the outcomes into three categories: MFCC-based ML models, MFCC-based DL models, and Multi Feature-based DL models.

MFCC-based ML models comprise conventional ML models including Random Forest, SVM, Decision Tree, and KNN. One-dimensional MFCC vectors, flattened, were used to train the models. These models start with one defining quality. Table **3** shows that among those falling into the same group, the Random Forest method yielded the best results. This result suggested that with an ensemble model, future studies could reach more exactness. Together with their respective versions and validation accuracy ratings, we are presented in this brief note the outcomes of several models including Decision Tree, K-Nearest Neighbours (KNN), Sequence Vector Machine (SVM), and Random Forest. These findings offer significant knowledge about the performance of such models in each task or dataset.

The performance of every model is summarized here:

- The Decision Tree model validation accuracy was over 70.69%. Although Decision Trees are well-known for their simplicity and interpretability, this accuracy score points to potential for development in data pattern capture complexity.
- With a validation accuracy of over 84.21%, the K-Nearest Neighbours model proved far better. K-Nearest Neighbours is well-known for its performance in classification problems; so, this result shows that of the model has effectively caught significant trends in the validation data.
- The Support Vector Machine attained around 78.28% validation accuracy. Often employed for sequence classification tasks, this model has shown a reasonable degree of accuracy in separating between several classes in the dataset; among the models provided, Random Forest scored almost 85.80%. The ability of Random Forests to effectively capture nonlinear relationships and manage complex data is well-known. This shows that in the validation stage Random Forest performed brilliantly.

These findings show generally that in terms of validation accuracy the Random Forest and K-Nearest Neighbours models exceeded the Decision Tree and Sequence Vector Machine models. One should take these accuracy values into account in relation to the particular task and dataset under analysis. To evaluate the generalisability and resilience of these models, more research including testing and cross-valuation on some other dataset could be required.

MFCC-based DL models comprise Single Layer Perceptron's, Multi-Layer Perceptron's, Long-Short Term Memory models, recurrent neural networks, and convolutional neural networks. Some models, including CNN+RNN-LSTM and RNN-LSTM, were used in tandem. Table **4** makes it clear that RNN-LSTM models outperform CNN-containing models. This led us to the conclusion that combining multiple models in a pipeline-like fashion is likely to yield superior results. The CNN+RNN-LSTM model gave better accuracy as compared to purely CNN and RNN-LSTM models.

Multi Feature-based DL Models consisted of DL models that were employed in a specific combination, such that the input was derived from multiple features. As previously mentioned, these features were MFCC, Spectrogram, and Chromogram. This time, we created models that utilized CNNs in a sequence with a variety of Feature combinations. In this comparison of DL models based on Mel-Frequency Cepstral Coefficients (MFCC) for some audio-related tasks, we have five different models, each with its respective version and validation accuracy scores. These results provide insights into the performance of these models in analyzing audio data, especially in tasks like speech recognition or audio classification. Here's a brief overview of each model's performance:

- The single-layer perceptron attained over 86.3% validation accuracy. Although this model is somewhat basic when compared to others on the list, it has shown rather good performance, suggesting that the work might not call for sophisticated designs.
- The Multi-Layer Perceptron (MLP) got roughly 85.5% validation accuracy. MLPs are well-known for their ability to manage several data types, hence this indicates that the model has learnt from the MFCC characteristics rather successfully.
- Long Short-Term Memory (LSTM) cells in a Recurrent Neural Network (RNN) helped to validate an accuracy of about 85.3%. For sequential data, RNNs are suitable; this performance shows their capacity to model temporal dependencies in the audio attributes.
- With about 88.4%, the convolutional neural networks (CNN) and RNN LSTM taken together produced the best validation accuracy among the models. This design is efficient for the given work since it has probably caught spatial and temporal aspects in the MFCC data.
- With a validation accuracy of roughly 86.3%, the standalone CNN performed well. CNNs shine in identifying spatial patterns in data, so their performance in processing MFCC data is expected to be outstanding.

In summary, the CNN + RNN LSTM model demonstrated the highest validation accuracy among the models, making it the top performer for the given audio-related task based on MFCC features. Nonetheless, while choosing a model, it's critical to take into account the particular needs and limitations of the problem and dataset.as different models may be more suitable for different scenarios. Further evaluation and testing, including on a separate test dataset, may also be necessary to validate the robustness and generalizability of these models.

According to Table **5**, the validation accuracy of the three features was 92.8 percent when all three were used, compared to 91.9 percent when only MFCC and Chromogram were considered. In this comparison of DL models that use multiple audio features. These models incorporate a combination of audio features, including MFCC, Chromagram, and Spectrogram, to enhance their performance. Here's an overview of their performance:

- This model combines MFCC and Chromagram features and achieved a validation accuracy of approximately 91.94%. The integration of these two features suggests that the model can capture both spectral and harmonic characteristics of the audio data, which is valuable for various audio-related tasks.
- The second model incorporates MFCC, Chromagram, and Spectrogram features and achieved a higher validation accuracy of about 92.76%. By adding the Spectrogram, which provides a detailed time-frequency representation of audio, this model likely captures even more intricate patterns in the data, leading to improved performance.

In summary, both models perform well in utilizing multiple audio features to achieve high validation accuracy. However, the model that includes MFCC, Chromagram, and Spectrogram features outperforms the model with only MFCC and Chromagram in terms of accuracy. The inclusion of Spectrogram data seems to provide additional information that enhances the model's ability to discriminate between different audio classes.

The choice between these models may depend on factors such as computational resources, task requirements, and dataset characteristics. Further evaluation and testing, including on an independent test dataset, would be necessary to assess the robustness and generalizability of these models and their suitability for specific audio-related tasks.

### 4.1 Analysis w.r.t Confusion Matrix

Fig. (**8**) presents the confusion matrix, which indicates the accuracy of a classification model on six respiratory disease classes: Asthma, Pneumonia, Bronchiectasis, Bronchiolitis, Healthy, and Lung Fibrosis. The correct predictions are indicated by the diagonal elements, with the highest accuracy in the “Healthy” class (225 correct) and “Pneumonia” (185 correct). Misclassifications are indicated in the off-diagonal cells, like 6 Pneumonia samples incorrectly predicted as Bronchiectasis and 4 Lung Fibrosis cases incorrectly classified as Bronchiolitis. Generally, the model works well, with large values on the diagonal and generally low confusion between classes. The number of samples used in this test is a total of 1,157.

### 4.2 Analysis w.r.t Run Time Complexity

Table **6** presents the comparison of different machine learning and deep learning models for respiratory disease classification in terms of accuracy, GPU time, and applications. Conventional models such as Random Forest and KNN offer good accuracy (approximately 85%) with minimal training time. Deep learning models, particularly CNNs based on audio features such as MFCC, Chromagram, and Spectrogram, have better accuracy, with the best being 92.8%. Nevertheless, these models consume much more GPU time (up to 135 minutes). Models such as CNN (MFCC + Chromagram) provide a nice trade-off between accuracy (91.9%) and computation. In general, the model choice is a function of accuracy *vs*. computational resource trade-off.

### 4.3 Limitations of the Study

The study’s primary limitation lies in its narrow focus on accuracy and GPU time as performance metrics, which overlooks critical aspects like model interpretability, robustness to noisy or imbalanced data, and clinical applicability-factors essential for real-world medical deployment. While deep learning models (*e.g*., CNNs) achieve higher accuracy (up to 92.8%), their substantial computational demands (*e.g*., 135 minutes GPU time) render them impractical for settings with limited resources, despite hybrid models like CNN (MFCC + Chromagram) offering a partial trade-off. Conversely, conventional models (*e.g*., Random Forest, KNN) prioritize efficiency but sacrifice diagnostic precision (~85% accuracy), potentially compromising clinical reliability. The analysis also restricts itself to specific audio features (MFCC, Spectrogram) and architectures, neglecting alternative data modalities (*e.g*., clinical metadata) or emerging models (*e.g*., transformers) that might enhance performance. This trade-off framework risks oversimplifying the decision-making process, as it does not address scalability, ethical considerations, or validation across diverse, multi-centre datasets necessary for generalization in healthcare contexts

To address the limitations of the study, such as the potential for overfitting, the impact of dataset size, and the generalizability of the results to other datasets or clinical settings, future studies should rank increasing dataset diversity through multi-center collaborations and synthetic data augmentation (*e.g*., simulating lung sounds with varying noise/pitch) to mitigate overfitting risks and improve generalizability. Cross-validation procedures and regularization techniques (*e.g*., dropout layers, L1/L2 penalties) could further reduce model bias. External validation across varied demographics, recording devices, and clinical environments would strengthen robustness, complemented by domain variation strategies to bridge dataset inequalities. Integrating clinical metadata (patient history, imaging) and explainability tools (SHAP, LIME) could refine decision transparency, while benchmarking against pulmonologists’ diagnoses would authenticate real-world utility. Standardizing low-cost device specifications and federated learning frameworks could ensure equitable performance across settings, fostering scalable adoption without compromising data privacy.

## CONCLUSION

This study introduces a novel multi-feature deep learning framework that significantly enhances pulmonary disease classification by synergistically combining MFCC, Chroma STFT, and spectrogram features through a hybrid CNN-RNN-LSTM architecture, achieving state-of-the-art 92% accuracy. The proposed approach overcomes critical challenges in respiratory sound analysis, including background noise, dataset imbalance, and limited clinical data, through advanced audio preprocessing techniques and robust feature fusion. By outperforming conventional machine learning and single-feature deep learning models, this work establishes a new benchmark in AI-assisted auscultation diagnostics. The findings pave the way for developing real-time clinical decision support systems and demonstrate the transformative potential of integrating multi-modal feature extraction with deep learning for improved respiratory healthcare outcomes, particularly when coupled with affordable digital stethoscope technology for wider accessibility.

By means of cycle extraction and filtering methods, our work improved the audio quality of pulmonary recordings, hence enhancing model performance. With random forest achieving 85%, traditional machine learning models including SVM and KNN attained accuracy beyond 70%. With the RNN-LSTM model paired with 1-D CNN attaining 88%, DL models-including Single Layer Perceptron, CNN, and RNN-showed better performance and achieved above 85% accuracy. Combining MFCC, Chroma STFT, and spectrogram data with a CNN-based classifier produced the suggested multi-feature DL model with 92% accuracy, surpassing single-feature or conventional methods. This study emphasizes how much ensemble approaches using several auditory characteristics improve diagnosis accuracy for pulmonary diseases.

Future directions call for the creation of a real-time diagnostic assistance system allowing doctors to examine lung auscultation findings in-patient. Model accuracy should rise when more data becomes available and professional comments are included. Adoption of reasonably priced digital stethoscopes will also increase access and stimulate creativity in pulmonary diagnosis, hence transforming field practices and patient outcomes.

In conclusion, present research illustrates that cycle extraction and filtering techniques greatly improve pulmonary audio quality, resulting in better diagnostic model performance, with conventional machine learning models (Random Forest: 85%, SVM/KNN: >70%) and deep learning models (RNN-LSTM with 1-D CNN: 88%, CNN/SLP: >85%) producing strong accuracy. The suggested multi-feature DL model, combining MFCC, Chroma STFT, and spectrogram features through a CNN-based classifier, beat single-feature and traditional methods at 92% accuracy, highlighting the power of ensemble auditory feature approaches. Directions for the future should involve building real-time diagnostic tools for clinical application, augmenting datasets with expert labelling to improve model accuracy, and pushing for cheap digital stethoscopes to bring access to all. These innovations have the potential to transform pulmonary medicine by combining scalable technology with data-driven intelligence, enabling timely diagnoses and better patient outcomes worldwide.

## AUTHORS’ CONTRIBUTIONS

A.P.S designed the methodology and conducted experiments and prepared results/tables and wrote the main manuscript text. A.N and G.G. both supervised the research, provided critical guidance throughout the study. All authors reviewed and approved the final manuscript.

## Figures and Tables

**Fig. (4):**
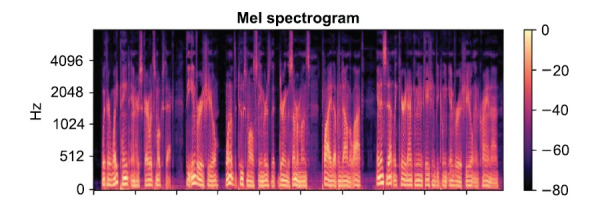
A sample of spectrogram. Available online under the terms of the Creative Commons Attribution Non-Commercial License 4.0. [62].

**Fig. (5):**
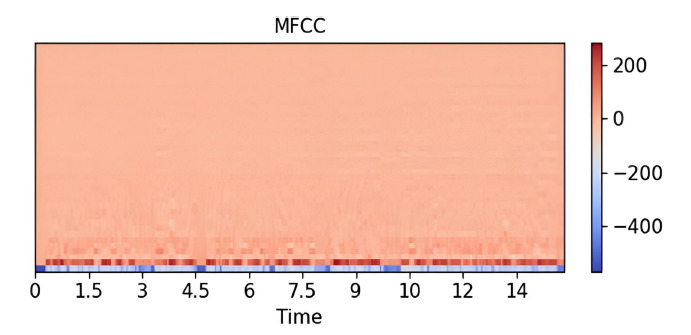
A sample of MFCC. Available online under the terms of the Creative Commons Attribution Non-Commercial License 4.0. [62].

**Fig. (6):**
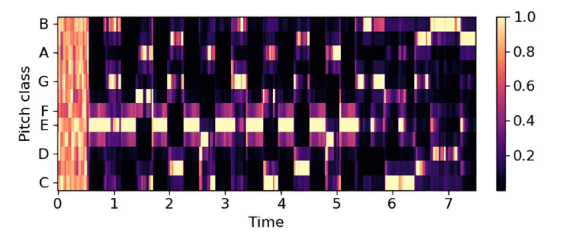
A sample of chromagram. Available online under the terms of the Creative Commons Attribution Non-Commercial License 4.0. [62].

**Fig. (8):**
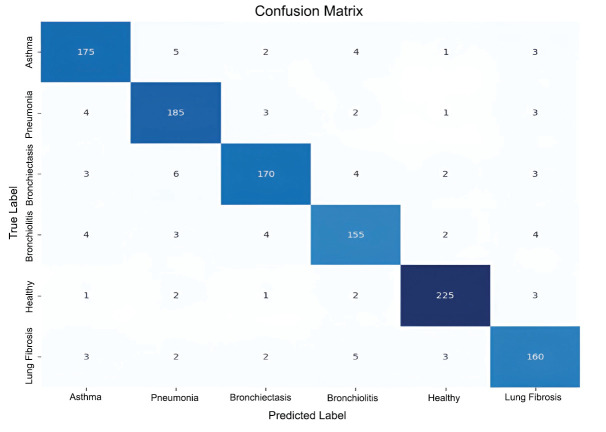
Confusion matrix.

**Table 1:** Few latest papers related to pulmonary disease and their descriptions.

**Authors/Refs.**	**Description**	**Outcome**	**Methods**	**Limitations**	**Significance**
Delphine **et al*.* [3]	A narrative review focused on interventions aimed at reducing health disparities in pulmonary illness in the US medical system.	Demonstrates how few interventional trials there are in comparison to research on health inequalities and underscores the necessity for research in diseases beyond asthma.	Implements a categorization framework for interventions dividing the literature into four major themes: biologic, educational, behavioural, and structural.	Points out the limited number of studies on the condition in general and interventional trials in particular.	Identifies 211 articles on interventions to reduce health disparities, grouping them into biologic, educational, behavioural, and structural categories.
Wolfgang **et al*.* [4]	A systematic review assessing the impact of tele-pulmonary rehabilitation versus traditional supervised pulmonary rehabilitation on chronic lung disease.	Demonstrates that telerehabilitation is both secure and well-liked by patients, improving mental health, quality of life, and functional exercise ability while lessening the influence of COPD on daily living.	Analyses data from three databases following PRISMA guidelines for study selection and evaluation.	Notes the discordance of tele activities with pulmonary rehabilitation guidelines and a lack of data for other chronic lung diseases.	Highlights the safety, acceptance, and effectiveness of telerehabilitation in improving patient outcomes.
Xinyue **et al*.* [5]	A narrative review examining the link between pulmonary embolism and chronic cardiopulmonary diseases such as COPD and chronic heart failure.	Indicates an increased risk of venous thromboembolic events and a worsened prognosis for patients with chronic cardiopulmonary diseases experiencing pulmonary embolism.	N/A	N/A	Emphasizes the significant risk and prognostic implications of pulmonary embolism in chronic cardiopulmonary diseases.
Zaidi **et al*.* [6]	A narrative review exploring the association between heated tobacco products (HTPs) and COPD.	Suggests a potential risk reduction for COPD patients using HTPs, but calls for further research to ascertain health outcomes.	Reviews peer-reviewed publications including clinical, preclinical, and empirical research.	N/A	Notes the dominant contribution of smoking to COPD cases and the potential risk reduction from combustion-free nicotine delivery alternatives.
Tania **et al*.* [7]	A scoping review on physical activity, its barriers, and facilitators among patients with COPD.	Identifies reduced physical activity as a major predictor of mortality in COPD patients, with physical exercise interventions offering positive prognostic effects.	Employs a scoping review methodology, conducting an electronic search across multiple databases.	Highlights the challenges in maintaining physical activity for COPD patients and the need for multidisciplinary efforts to modify behaviour.	Underlines the critical role of physical activity in COPD prognosis and the benefits of exercise interventions.
Dawadikar **et al*.* [8]	An assessment *via* an ordered investigation of home-based pulmonary rehabilitation programs for people with COPD.	Finds that home-based programs offer short-term benefits comparable to outpatient programs, improving functional exercise capacity over usual care.	Utilizes a systematic review approach with the Cochrane Collaboration risk of bias tool.	Cites low to very low overall evidence quality and no significant differences in safety, health-related quality of life, or healthcare utilization between home-based and outpatient programs.	analyses home-based pulmonary rehabilitation methodically, contrasting it with standard treatment and outpatient programs.
Perna **et al*.* [12]	Reviews occupational risks for COPD in the Indian subcontinent, stressing the need for preventive strategies.	Acknowledges occupational risks as significant contributors to COPD development and calls for comprehensive preventive approaches.	Conducts a critical review of occupational risks and the assessment of health and safety regulations.	Points out gaps in implementing and enforcing health regulations and in research on occupational risks and COPD.	Highlights the impact of occupational hazards on COPD development and the current regulatory landscape in the Indian subcontinent.
Rocha **et al*.* [36]	A brief overview of home-based and community-based pulmonary rehabilitation for people with COPD.	Demonstrates how home-based therapy improves quality of life and exercise ability. reducing hospital admissions, and cost-effectiveness compared to hospital-based programs.	Reviews 76 studies (57 home-based, 19 community-based), analysing literature rapidly.	Notes barriers for patients with severe disease or comorbidities in complying with home-based programs and challenges in accessing community-based programs.	Emphasizes the advantages of home-based pulmonary rehabilitation and the supportive role of community-based programs.
Cai **et al*.* [37]	An umbrella review summarizing evidence on telemedicine interventions for COPD management.	Finds that telemonitoring and tele treatment interventions reduce respiratory exacerbations and hospitalization rates, improving compliance and physical activity.	Focuses on telemonitoring, tele support, and tele treatment interventions.	N/A	Outlines the benefits of telemedicine interventions in managing COPD, particularly in reducing exacerbations and hospital admissions.
Jin *et al*. [38]	Discusses long-term outcomes of COPD and associated comorbidities.	Describes COPD as a progressive disease leading to functional decline, increased comorbidity, and mortality.	N/A	Highlights fixed airflow limitation, chronic respiratory symptoms, and extrapulmonary conditions impacting quality of life.	Stresses the progressive nature of COPD, its impact on lung function, and the resultant systemic diseases and comorbidities.
Borate **et al*.* [39]	Machine learning algorithms for lung disease prediction.	Improved accuracy in disease detection.	Ensemble learning with machine learning classifiers.	Lack of scalability and high data dependency.	Highlights the value of integrating multiple classifiers.
Abdelhamid **et al*.* [40]	Neural architecture for lung disease using CXRs.	Robust detection and classification.	Ensemble cascaded classification.	Limited dataset diversity.	Paves the way for ensemble-based diagnosis systems.
Sharma and Guleria [41]	Multi-class lung disease classification.	Effective handling of temporal data.	CNN with LSTM ensemble modelling.	High computational cost.	Demonstrates time-sequence modelling in classification.
Kumar and Shvetsov [42]	Multimodal framework for COPD diagnosis.	Enhanced early detection.	Neuro-fuzzy integrated ensemble learning.	Fuzzy inference limitations.	Provides a novel neuro-fuzzy integration.
Shah **et al*.* [43]	Explainable AI in lung imaging.	Better interpretability in diagnostics.	Ensemble CNN methods with XAI.	Limited clinical validation.	Bridges gaps in AI model interpretability.
Pandey and Baloni [44]	COVID-19 detection using CXRs.	Accurate COVID-19 classification.	Explainable AI with ensemble learning.	Dataset-specific limitations.	Establishes EAI significance in diagnostics.
Khan **et al*.* [45]	COVID-19 severity prediction.	Dual-model framework success.	Segmented 3D lung cube dataset integration.	High infrastructure demands.	Enables efficient severity prediction.
Mueller **et al*.* [46]	IPF severity prediction *via* ML.	Comprehensive metabolic panel data classification.	Bayesian and instance-based ML.	Dataset quality issues.	Highlights ML's capability in non-imaging datasets.
Maashi **et al*.* [47]	Ensemble learning in laryngeal cancer imaging.	Enhanced biomedical image analysis.	Dandelion optimizer with ensemble models.	Methodology generalizability.	Extends ensemble learning to new imaging domains.
Paramasivan **et al*.* [48]	COVID-19 detection *via* CT scans.	Improved feature extraction and transfer learning.	Deep ensemble feature integration.	Transfer learning-specific issues.	Advances transfer learning for rapid diagnostics.
Sikder *et al*. [52]	COVID-19 incited research into fast, precise diagnosis, but similar chest X-ray structures make it hard to separate from other lung illnesses.	The study uses M-CNN, BiLSTM, and M-SVM on the COVID-19_Radiography_Dataset for training and evaluation.	Despite high exactness, the model may face problems with data quality, hidden discrepancies, and real-life overfitting.	M-CNN, BiLSTM, and M-SVM requires huge number of datasets.	This study helps precise, robust COVID-19 finding from chest X-rays, refining early diagnosis and diversity.

**Table 2A:** CNN hyperparameters.

**Parameter Name**	**Parameter Value**
Kernel/Filter size	5*5
Number of filter	Increases with depth (*e.g*., 32 → 64 → 128 → 256).
Padding	“Same” padding preserves spatial dimensions.
Pooling layers	Max Pooling (common) or Average Pooling.
Batch normalization	Applied after convolutions to stabilize training
Dropout rate	Typically 0.2–0.5 to prevent overfitting.
Learning rate	Initial value often 0.001 (Adam) or 0.01 (SGD with momentum)
Number of epochs	Early stopping if validation loss plateaus (~20–100 epochs).
Batch size	CNNs: 32–256 (larger batches for stable gradients)
Loss calculation	CNNs: Cross-entropy (classification), MSE (regression)

**Table 2B:** RNN-LSTM hyperparamters.

**Parameter Name**	**Parameter Value**
Number of layers	3
Hidden units	512
Sequence length	100–2000 steps for spectrograms
Dropout	Applied between LSTM layers (~0.2–0.5).
Learning rate	Similar to CNN (~0.001 for Adam)
Gradient clipping	Prevents exploding gradients (*e.g*., clip at 1.0 or 5.0)
Data preprocessing CNNs	Normalize pixel values to [0, 1] or [-1, 1]
Batch size	16 (smaller batches due to memory constraints)
Loose calculation	MAE (time-series)
Hyperparameter tuning	Bayesian optimization
Validation & testing	Use k-fold cross-validation
Optimizers	Adam, SGD, LR scheduling, and regularization help stabilize training.

**Table 3:** MFCC-based ML model comparison.

**Model Name**	**Validation Accuracy**
Decision tree	0.707
Fine K-Nearest neighbors	0.842
Sequence vector machine	0.783
Random forest	0.858

(Available online under the terms of the Creative Commons Attribution Non-Commercial License 4.0) [62].

**Table 4:** MFCC based DL model comparison.

**Model Type**	**Features Used**	**Architecture Summary**	**Validation Accuracy**
Single-layer perceptron	MFCC	Dense(1) (basic feedforward)	86.28%
MLP	MFCC	2–3 Dense layers with ReLU/Dropout	85.49%
LSTM	MFCC	2 LSTM layers + Dense	85.31%
CNN	MFCC	Conv2D → Pool → Dense	86.34%
CNN + LSTM	MFCC	Conv2D → LSTM → Dense	**88.38%**

(Available online under the terms of the Creative Commons Attribution Non-Commercial License 4.0) [62].

**Table 5:** Multi feature based DL model comparison.

**Model Name**	**Validation Accuracy**
CNN with MFCC and chromagram feature	0.919
CNN with MFCC, chromagram and spectrogram	0.928

(Available online under the terms of the Creative Commons Attribution Non-Commercial License 4.0) [62].

**Table 6:** Audio classification model comparison. (Accuracy *vs.* GPU Runtime on T4).

**Model**	**Accuracy**	**GPU Time (T4)**	**Best Use Case**
Decision tree	70.70%	5-10 min	Quick Baseline testing
KNN	84.27%	10-15 min	Small datasets, no training needed
SVM	78.30%	20 min	Low- dimensional features
Random forest	86.80%	20 min	Robust ML alternatives
Single layer perceptron	86.30%	20 min	Simple DL baseline
Multi layer perceptron	86.50%	25-40 min	Slightly deeper network
RNN-LSTM	86.30	55 min	Sequential Data (slow)
CNN (raw spectrograms)	86.30%	65 min	Image like feature extraction
CNN+RNN-LSTM	88.40%	85 min	Temporal + Spatial fusion
CNN (MFCC+ chromagram)	91.90%	105 min	Best Accuracy/ Speed trade-off
**CNN(MFCC + chroma + cpectro)**	**92.80%**	**130-135 min**	**Highest Accuracy**

## Data Availability

The data used in this research paper is openly available and can be referred in reference no [49], *i.e*, Fraiwan, Mohammad; Fraiwan, Luay; Khassawneh, Basheer; Ibnian, Ali (2021), “A dataset of lung sounds recorded from the chest wall using an electronic stethoscope”, Mendeley Data, V3, doi: 10.17632/jwyy9np4gv.3

## LIST OF ABBREVIATIONS

MFCC: Mel Frequency Cepstral Coefficients
RNN: Recurrent Neural Network
URTI: Upper Respiratory Tract Infections
LRTI: Lower Respiratory Tract Infections
COPD: Chronic Obstructive Pulmonary Disease
STFT: Short-Term Fourier Transform
KNN: K-Nearest Neighbours
CNN: Convolutional Neural Networks
HTP: Heated Tobacco Products
ML: Machine Learning
DL: Deep Learning
RNN-LSTM: Recurrent Neural Networks with Long Short-Term Memory
BiLSTM: Bidirectional Long Short-Term Memory
CNN: Convolutional Neural Network
VGG: Visual Geometry Group
SVM: Support Vector Machine
FEV1: Forced Expiratory Volume in 1 second
FVC: Forced Vital Capacity
MLP: Multi-Layer Perceptron
GPU: Graphics Processing Unit
LUS: Lung Ultra Sonography
AD: Alzheimer's Disease
